# Hotspots Evolution and Cutting‐Edge Trends: A Bibliometric Analysis of Autophagy in Myocardial Infarction Studies From 2007 to 2025

**DOI:** 10.1155/cdr/9777688

**Published:** 2026-05-14

**Authors:** Xiao-lin Li, Jin-wen Wu, Zeng-dai-quan Ke, Yuan-li Hu, Hang Jiang, Ming-tai Chen, Han-yu Hu, Zhong-jing Hu, Yuan-yuan Li, Gang Luo, Meng-nan Liu

**Affiliations:** ^1^ Department of Cardiology, The Affiliated Traditional Chinese Medicine Hospital, Southwest Medical University, Luzhou, Sichuan, China, swmu.edu.cn; ^2^ School of Integrated Traditional Chinese and Western Medicine, Southwest Medical University, Luzhou, Sichuan, China, swmu.edu.cn; ^3^ Faculty of Chinese Medicine and State Key Laboratory of Quality Research in Chinese Medicine, Macau University of Science and Technology, Macau, China, must.edu.mo; ^4^ Department of Cardiology, Shenzhen Traditional Chinese Medicine Hospital, Shenzhen, Guangdong, China, szszyy.cn; ^5^ Guangdong-Macao ln-Depth Cooperation Zone in Hengqin, Chinese Medicine Guangdong Laboratory (Hengqin Laboratory), Zhuhai, Guangdong, China; ^6^ State Key Laboratory of Traditional Chinese Medicine Syndrome, State Key Laboratory of Dampness Syndrome of Chinese Medicine, Guangdong Provincial Key Laboratory of Chinese Medicine for Prevention and Treatment of Refractory Chronic Diseases, Guangdong Provincial Hospital of Chinese Medicine, Guangdong Provincial Academy of Chinese Medical Sciences, The Second Affiliated Hospital of Guangzhou University of Chinese Medicine, Guangzhou, China, gzucm.edu.cn

**Keywords:** autophagy, bibliometric analysis, cardioprotection, myocardial infarction

## Abstract

**Background:**

Myocardial infarction (MI), a severe cardiovascular disorder, has a well‐established association with autophagy and disease progression. However, bibliometric analyses of this relationship remain limited.

**Methods:**

All literature related to autophagy in MI published between 2007 and 2025 was collected from the Web of Science Core Collection (WoSCC) and Scopus. The final database search was conducted on December 31, 2025. HistCite Pro, CiteSpace, VOSviewer, SCImago Graphica, and Origin were used for bibliometric analysis and visualization.

**Results:**

This search identified 649 publications on autophagy in MI. China was the most productive country, with Harbin Medical University as the leading institution. Researchers from Japan also made substantial contributions. *Biochemical and Biophysical Research Communications* published the highest number of articles. Sadoshima, Junichi was the most active researcher in this field. Current research hotspots include “oxidative stress” (the most recent focus) and “survival” (with the strongest citation bursts).

**Conclusions:**

This bibliometric analysis synthesizes research trends in autophagy‐related MI studies from 2007 to 2025. Future research should prioritize elucidating the mechanistic roles of autophagy in MI, particularly the interplay between mitophagy and the NLRP3 (NOD‐like receptor family pyrin domain‐containing Protein 3) pathway, to facilitate the development of more effective clinical interventions.

## 1. Introduction

Myocardial infarction (MI) is characterized by ischemic injury and necrosis caused by a severe reduction or complete cessation of coronary blood flow [1]. This condition occurs when the coronary arteries are occluded by atherosclerotic plaque rupture, thrombosis, or vasospasm, resulting in an interrupted oxygen and nutrient supply to cardiomyocytes. Such ischemia disrupts cellular energy metabolism and initiates complex pathophysiological cascades, including mitochondrial dysfunction, calcium overload, and accumulation of reactive oxygen species (ROS) [2], and activation of regulated cell death pathways such as necrosis, apoptosis, and autophagy. These processes ultimately lead to irreversible cardiomyocyte damage, followed by inflammatory infiltration and tissue repair responses [3].

Autophagy, a conserved catabolic process, plays a dual role in MI by maintaining cardiomyocyte homeostasis through the clearance of damaged proteins and organelles. Myocardial ischemia activates autophagy [4], promoting the degradation of misfolded proteins and dysfunctional mitochondria [5]. During reperfusion, controlled autophagy removes ischemia‐induced cellular debris, thereby limiting infarct size [4]. The regulation of autophagy involves multiple signaling pathways, such as the AMPK/mTOR/ULK1 axis [6] and Beclin‐1 complex formation [7], which collectively determine autophagic flux. Beyond cell survival, autophagy also regulates inflammation and fibrosis during post‐MI cardiac remodeling [8]. However, excessive autophagy can exert detrimental effects. In the late phase of MI, dysregulated autophagy causes aberrant degradation of essential cellular components, such as functional mitochondria and contractile proteins, accelerating cardiomyocyte apoptosis and contractile dysfunction. This pathological process depletes cellular resources and may exacerbate autophagosome accumulation, ultimately counteracting cardioprotective effects, expanding infarct size, and aggravating adverse cardiac remodeling. Given its pivotal role in ischemia/reperfusion injury, elucidating the regulatory mechanisms of autophagy may inform the development of novel therapeutic strategies with clinical translational potential [5].

This study differs from previous bibliometric analyses concerning autophagy and cardiovascular diseases [9] by explicitly confining its scope to the specific domain of “autophagy and myocardial infarction”. Through quantitative analysis of literature within this field, we have not only systematically identified core research hotspots but also further explored the implications of these findings for guiding clinical research directions and drug development strategies in MI.

To address this gap, this study integrated literature retrieved from the Web of Science Core Collection (WoSCC) (2007–2025) and the Scopus database. Employing multiple analytical tools, it examined publication trends, collaborative networks, influential contributors, journal distribution, and keyword‐based thematic evolution. By focusing on MI, this study is aimed at delineating the unique knowledge structure, evolving research hotspots, and emerging directions within autophagy‐related MI research, thereby complementing and extending existing bibliometric studies in the cardiovascular field.

## 2. Materials and Methods

### 2.1. Data Acquisition and Search Strategy

**Figure 1:**
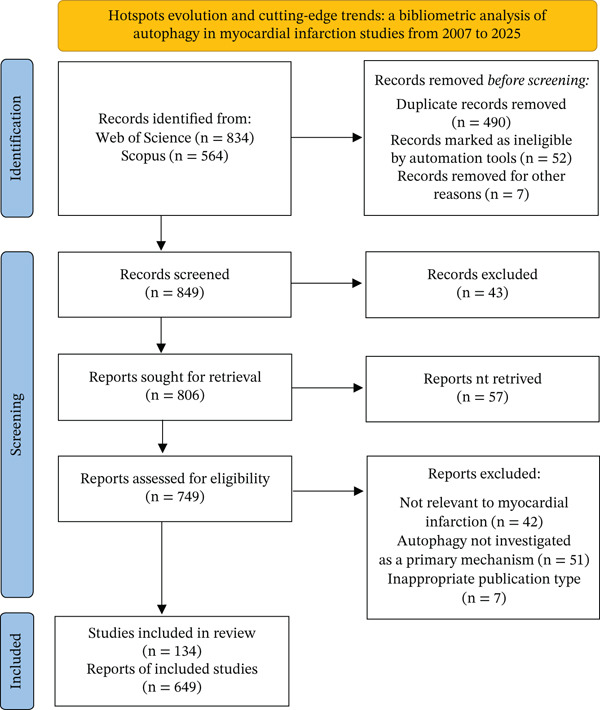
Flowchart of this bibliometric study.

A systematic literature search was conducted in the WoSCC and Scopus on 2026 to identify publications related to MI and autophagy. The search was performed within the Science Citation Index Expanded (SCI‐EXPANDED), and the final retrieval was completed on December 31, 2025 The search strategy employed the following Boolean query: TS = (autophagy OR cell autophagy OR macroautophagy OR mitophagy OR microautophagy OR chaperone‐mediated autophagy OR autophagosome OR autophagic flux OR lysosomal degradation) AND TS = (Myocardial Infarction OR myocardial infarction OR Infarction, Myocardial OR heart attack OR Infarction, Myocardial OR cardiac ischemia OR Myocardial Infarct OR myocardial ischemia OR MI OR acute myocardial infarction OR AMI OR ST‐segment elevation myocardial infarction OR STEMI OR non‐ST‐segment elevation myocardial infarction OR NSTEMI). Search strategies were illustrated in Figure 1. Only English‐language articles and review articles were included in the analysis. Other document types, including meeting abstracts, editorial materials, letters, corrections, and notes, were excluded. “Early Access” publications were also eligible for inclusion, provided they met all other criteria. The retrieved records were exported in plain text format for further processing. Following the search, data processing was conducted to create a unified analysis dataset. Records retrieved from WoSCC and Scopus were first merged into a combined dataset. Subsequently, deduplication and data harmonization were performed using thebibliometrixpackage (R and Biblioshiny interface). The deduplication process relied primarily on DOI matching, followed by title and author matching. This process effectively consolidated early‐access versions with their corresponding final published records, ensuring a nonredundant corpus. As this study exclusively involved secondary analysis of published literature without any original human or animal data, institutional review board approval was not required.

### 2.2. Data Analysis

Referring to previous bibliometric studies [10, 11], the bibliometric analysis employed multiple complementary analytical approaches to comprehensively characterize publication patterns and knowledge structures in this field. The bibliometric analysis employed multiple complementary analytical approaches. Publication trends were quantitatively analyzed and visualized using Origin 2024. For network analysis and visualization, VOSviewer (Version 1.6.18) was utilized to construct country and institutional collaboration networks, journal cocitation networks, author collaboration networks, and keyword co‐occurrence networks. Prior to analysis in CiteSpace, all records were preprocessed in NoteExpress to standardize the database source field to “WOS” and were exported in the compatible format. According to prior bibliometric methodology literature [12, 13], CiteSpace (Version 6.2.R4) was applied for advanced analyses, including dual‐map overlay of journals, reference cocitation analysis with timeline visualization to track research evolution, and burst detection to identify emerging trends. Geographic distribution patterns of research output were mapped using SCImago Graphica (Version 1.0.42), and international collaboration patterns were further examined using an online bibliometric platform (https://bibliometric.com/app). This multitool approach ensured comprehensive coverage of both quantitative publication metrics and qualitative network relationships within the research domain.

For network construction and visualization, minimum thresholds were predefined to balance coverage and interpretability and to reduce noise from sporadically occurring nodes. Countries, institutions, and authors were included in coauthorship networks only if they met a minimum publication threshold, ensuring that stable and representative collaboration patterns were captured. Keyword co‐occurrence analysis applied a minimum occurrence threshold to focus on recurrent, thematically meaningful terms and to identify core research topics and trends. In CiteSpace, clustering was performed using the default log‐likelihood ratio (LLR) algorithm to generate statistically significant and interpretable thematic clusters. All parameters were specified a priori and applied consistently. In VOSviewer, the minimum number of publications was set to ≥ 7 for countries, ≥ 5 for institutions, ≥ 8 for citing journals, and ≥ 20 for cocited journals. Author analyses applied thresholds of ≥ 4 for author coauthorship network visualization and ≥ 5 for author overlay visualization. Keywords were included when they occurred at least five times. In CiteSpace, time slicing was set to 2007–2025 (1 year per slice). Authors with ≥ 25 citations and ≥ 25 cocitations were included in the author cocitation network, among whom the Top 30 most cocited authors were highlighted for visualization. A scale factor of 25 was applied to references and keywords. Sensitivity analyses yielded comparable structures, indicating robust results. TGCS (Total Global Citation Score) reflects a publication′s global citation impact across Web of Science and Scopus, whereas TLCS (Total Local Citation Score) indicates its citation influence within the autophagy‐MI dataset.

## 3. Results

### 3.1. Analysis of Publication Volume and Trend

**Figure 2:**
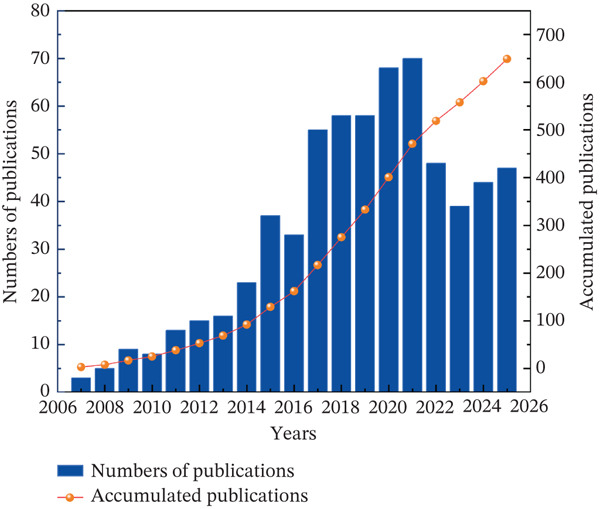
The annual number and the cumulative number of publications.

A total of 649 articles were retrieved from the WoSCC and Scopus database between 2007 and 2025, and as demonstrated in Figure 2, the publication trend revealed three distinct phases. The first phase (2007–2016) showed steady growth, with annual publications increasing from 1 in 2007 to 32 in 2016, though the overall output remained relatively low. The second phase (2017–2021) showed rapid expansion, evidenced by a steep slope in the trend line with annual publications rising from 55 to 69. The third phase (2022–2025) was characterized by fluctuations in publication output, with an overall stable trend rather than a sustained decline.

### 3.2. The Most Productive Countries

**Figure 3:**
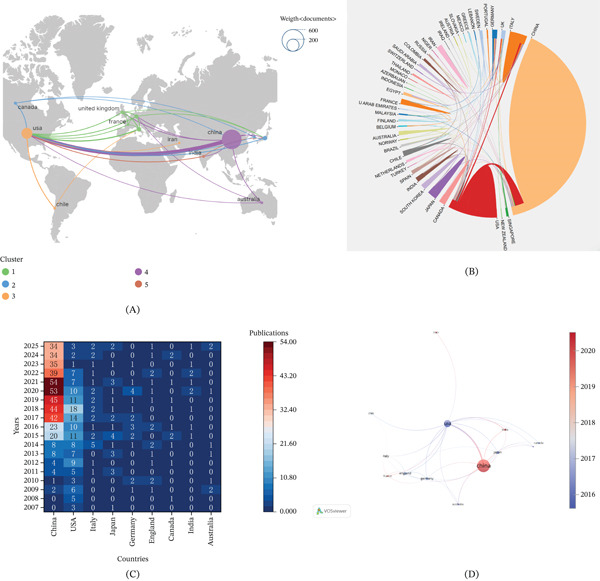
Visualization of countries. (A) The network visualization map of country coauthorship analysis. (B) The geographic distribution map based on the total literature of different countries/regions. (C) The overlay visualization map of country coauthorship analysis. The node color reflected the corresponding average appearing year (AAY) according to the color gradient in the lower right corner. (D) Hotmap of national publications.

Research on autophagy in MI involved 45 countries, as supported by Figure 3A. As shown in Table 1, China ranked first with 450 publications (63.38% of total output), followed by the United States with 139 publications (19.58%). Italy (*n* = 25, 3.52%), Japan (*n* = 24, 3.38%), and Germany (*n* = 19, 2.68%) ranked third to fifth, respectively. In terms of citation impact, China and the United States also showed high TLCS (642 and 529) and TGCS (20,949 and 15,524). Notably, Japan exhibited a relatively high TLCS (187) in relation to its publication volume. Figure 3B shows the international collaboration network, with collaborative links mainly concentrated in East Asia, North America, and Europe. China and the United States occupy central positions in the network. Temporal overlay analysis, as evidenced by Figures 3C,D indicates an increase in publication activity from China in recent years, alongside sustained contributions from Europe and North America.

**Table 1:** Top 10 active countries/regions.

Rank	Country/region	Publications	Percent	TLCS	TGCS
1st	China	450	63.38%	642	20,949
2nd	United States	139	19.58%	529	15,524
3rd	Italy	25	3.52%	114	2838
4th	Japan	24	3.38%	187	2566
5th	Germany	19	2.68%	92	2930
6th	UK	16	2.25%	28	1661
7th	Canada	11	1.55%	17	842
8th	India	10	1.41%	0	363
9th	Chile	8	1.13%	27	927
10th	Australia	8	1.13%	3	147

### 3.3. The Most Productive Institutions and Collaborations

**Figure 4:**
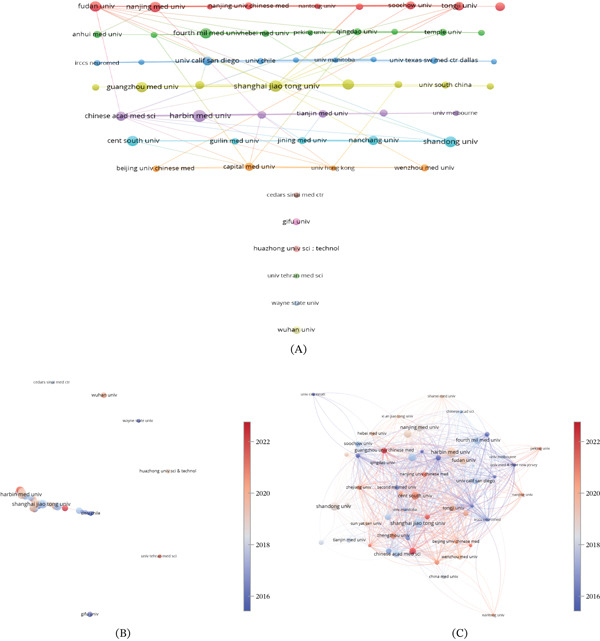
Visualization of institutions. (A) The network visualization map of journal coauthorship analysis. (B) The network visualization map of institutions coauthorship analysis. (C) The overlay visualization map of institutions citation analysis.

Since 2007, a total of 831 institutions have published research on autophagy in MI, with 56 institutions continuing to contribute to this field (Figure 4A,B). The Top 10 institutions are listed in Table 2. In terms of publication volume, the leading five institutions are Harbin Medical University (*N* = 21), Shanghai Jiao Tong University (*N* = 20), Shandong University (*N* = 18), Fudan University (*N* = 17), and Nanjing Medical University (*N* = 17). Based on total citation counts, the three most influential institutions are Rutgers Medical School of New Jersey (TGCS = 1980), the Italian IRCCS Institute of Neurological Sciences (TGCS = 1577), and the University of Washington (TGCS = 1409). Regarding institutional citation impact (citations per paper), the Top 3 institutions were Rutgers New Jersey Medical School (*n* = 396), IRCCS Neurological Institute of Italy (*n* = 262.8), and the University of Washington (*n* = 352.2). Institutional collaboration network analysis (Figure 4C) reveals that Harbin Medical University, Shanghai Jiao Tong University, and Shandong University form significant collaborative nodes within China, whereas the University of Texas Southwestern Medical Centre in Dallas occupies a central position within the US network. Multiple institutions serve as bridges in global collaborations.

**Table 2:** Top 10 most active institutions and cited institutions.

Rank	Most active institutions				Most cited institutions			
	Institutions	Publications	TLCS	TGCS	Institutions	Publications	TLCS	TGCS
1st	Harbin Med Univ	21	38	723	Rutgers New Jersey Med Sch	5	102	1980
2nd	Shanghai Jiao Tong Univ	20	52	1264	IRCCS Neuromed	6	100	1577
3rd	Shandong Univ	18	13	415	Univ Washington	4	12	1409
4th	Fudan Univ	17	33	952	China Med Univ	7	16	1326
5th	Nanjing Med Univ	17	26	474	Qingdao Univ	9	13	1289
6th	Cent South Univ	16	10	619	Shanghai Jiao Tong Univ	20	52	1264
7th	Fourth Mil Med Univ	16	35	1,051	Univ Texas SW Med Ctr Dallas	7	39	1226
8th	Guangzhou Med Univ	16	38	657	Univ Calif San Diego	12	64	1208
9th	Southern Med Univ	16	31	461	Albert Einstein Coll Med	4	16	1183
10th	Chinese Acad Med Sci	14	20	847	Soochow Univ	11	42	1135

### 3.4. Journal Analysis

**Figure 5:**
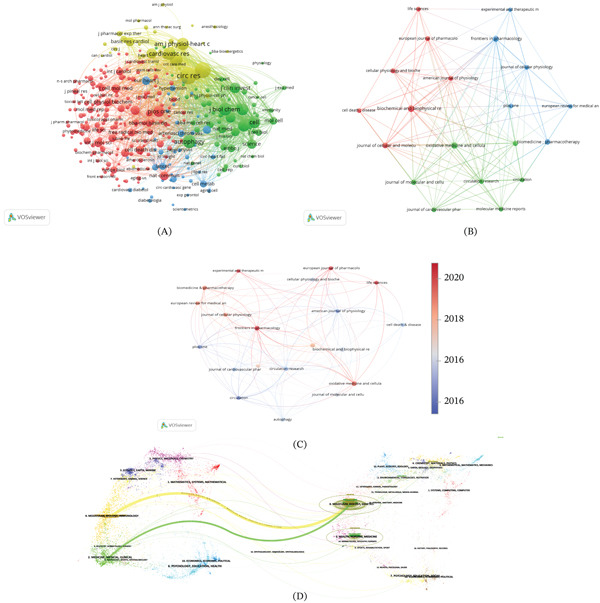
Visualization of journals. (A) The network visualization maps of cocitation journals. (B) The network visualization maps of citing journals. (C) The overlay visualization map of journal citing journals. (D) The dual‐map overlay of journals from 2007 to 2025.

A total of 649 publications on autophagy in MI were distributed across 280 journals, as shown in Figure 5A. Among them, 20 journals showed sustained publication activity during the study (Figure 5B,C). As summarized in Table 3, the Top 5 journals by publication count were *Biochemical and Biophysical Research Communications (* *N* = 17), *Oxidative Medicine and Cellular Longevity* (*N* = 15) (*Oxidative Medicine and Cellular Longevity* was indexed in WoSCC during the study period, although it was subsequently discontinued from SCIE in 2025), *Frontiers in Pharmacology* (*N* = 14), and *Biomedicine & Pharmacotherapy* (*N* = 12). Among the most productive journals, *Circulation Research* (IF = 16.2) exhibited the highest impact factors, and the majority of these journals were ranked in JCR Q1–Q2. In terms of citation performance, *Circulation Research* accumulated the highest total citations (TGCS = 2325), followed by *Cellular Physiology and Biochemistry* (TGCS = 1701) and *Journal of Molecular and Cellular Cardiology* (TGCS = 1435). The colored trajectories represent citation linkages, with citing journals positioned on the left and cited journals on the right (Figure 5D). A predominant citation pathway was identified, where publications in molecular/biology/genetics journals were primarily cited by studies in molecular/biology/immunology domains. Notably, journals covering physics/materials/chemistry and pharmacology/medicine/clinical disciplines also participated in autophagy research related to MI, demonstrating a confluent pattern of interdisciplinary integration.

**Table 3:** Top 10 most active journals.

Rank	Journals	Country	Publications	IF (2025)	JCR (2025)	TGCS
1st	Biochemical and Biophysical Research Communications	United States	17	1.84	Q4	688
2nd	Oxidative Medicine And Cellular Longevity	United States	15	0.0	Q2	704
3rd	Frontiers In Pharmacology	Switzerland	14	4.8	Q1	503
4th	Biomedicine & Pharmacotherapy	France	12	7.5	Q1	630
5th	Journal of Cellular and Molecular Medicine	United Kingdom	12	4.2	Q2	736
6th	Autophagy	United States	11	14.3	Q1	1332
7th	Journal of Molecular and Cellular Cardiology	United States	11	4.7	Q1	1435
8th	Cellular Physiology and Biochemistry	Switzerland	10	2.0	Q3	1701
9th	Circulation Research	United States	10	16.2	Q1	2325
10th	European Journal of Pharmacology	Netherlands	10	4.7	Q1	381

### 3.5. Science Overlay Maps and Interdisciplinary Connections

**Figure 6:**
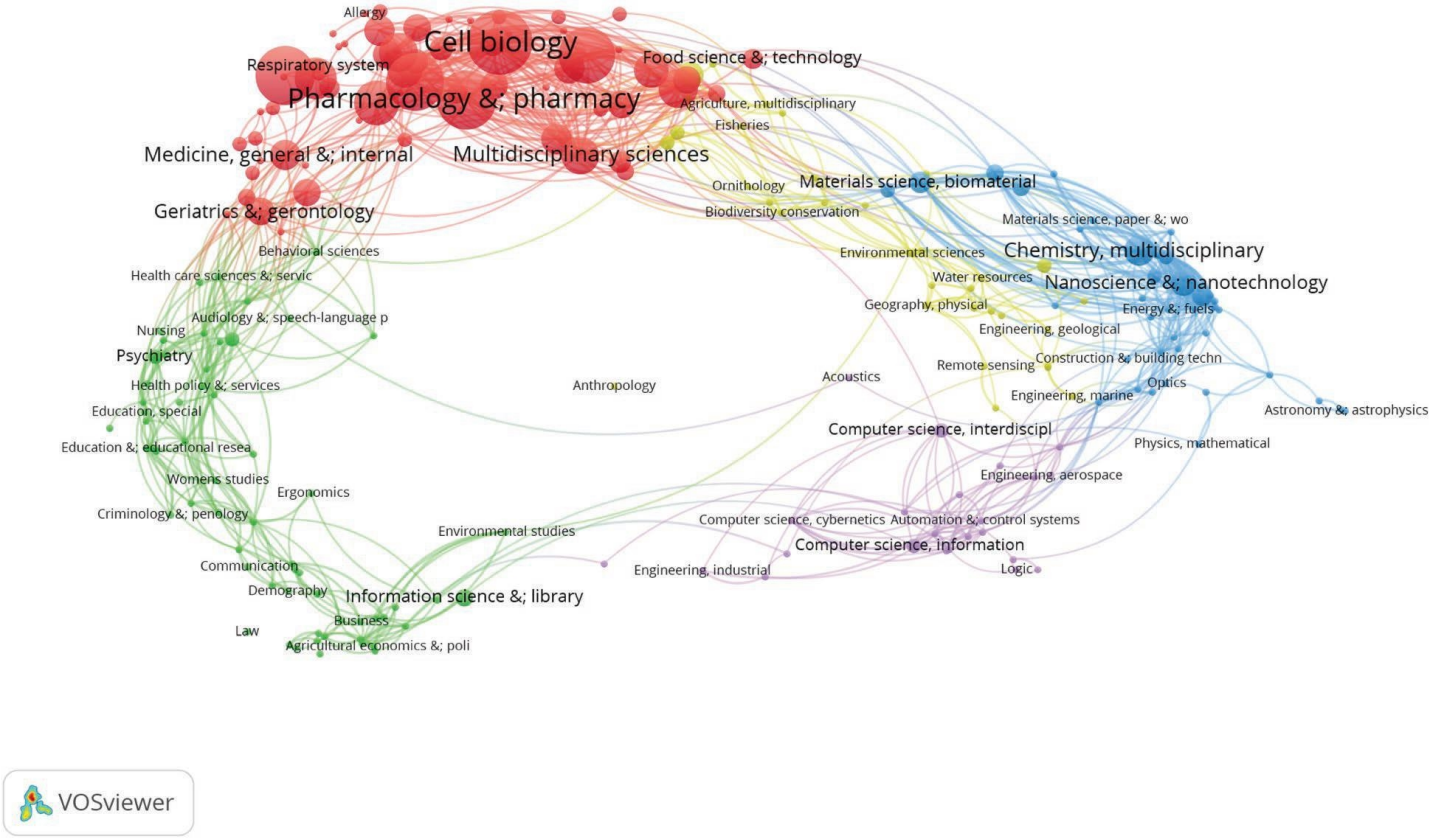
Science overlay maps.

Scientific overlay mapping was applied to examine interdisciplinary linkages in autophagy related MI research. Following the framework proposed by Leydesdorff and Rafol and further developed by Rafols and Meyer, science overlay maps were constructed based on a global map of science using aggregated journal citation data from 2007 to 2025. As visualized in Figure 6. The analysis indicates that the literature is primarily situated within the biological and medical sciences, specifically cardiovascular systems, cell biology, and physiology. Cross‐disciplinary associations with applied physics, computer science, statistics, and chemistry are also observed, which may reflect a degree of methodological and conceptual overlap across these fields, suggesting a certain level of similarity and complementarity and indicating potential for innovation.

### 3.6. Influential Authors and Cocitation Analysis

**Figure 7:**
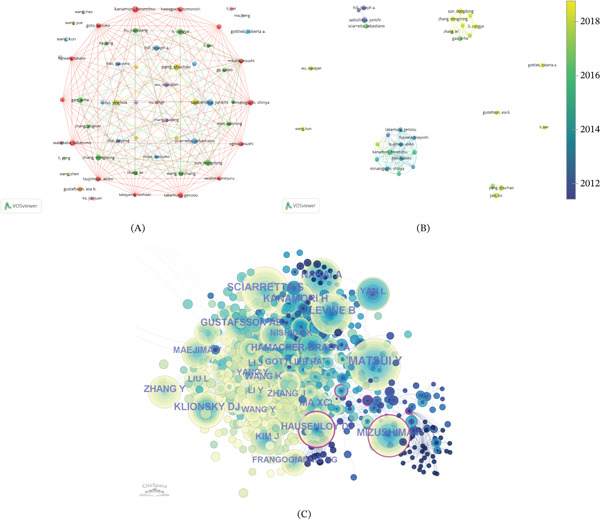
Visualization of authors. (A) The network visualization map of authors coauthorship analysis. (B) The overlay visualization map of authors coauthorship analysis. (C) Top 30 most cocited and cited authors.

A total of 4120 authors contributed to research on the autophagy mechanisms of MI. As shown in Table 4, among the Top 10 most prolific authors, key contributors included Sadoshima, Junichi (*N* = 10, TGCS = 2498), Kanamori, Hiromitsu (*N* = 9, TGCS = 888), and Pang, Shuchao (*N* = 9, TGCS = 99). Notably, some authors with fewer publications, such as Del Re, Dominic P., and Zhai, Peiyong, exhibit higher average citation rates, demonstrating high visibility of their individual contributions. As depicted in Figure 7A, the coauthor network analysis encompasses 58 authors meeting the minimum threshold of four publications, distributed across 18 clustered research groups. The red cluster, predominantly composed of Japanese researchers, exhibits the strongest network connectivity and highest publication output. Despite close collaboration within clusters, intercluster connections remain relatively limited. The overlay visualization, as illustrated in Figure 7B, reflects both long‐standing contributors and more recently active authors. In addition, cocitation analysis, as shown in Figure 7C, identified Matsui, Y. as the most frequently cocited author, whereas Mizushima, N. showed the highest betweenness centrality, suggesting a key bridging role in the cited‐author network.

**Table 4:** Top 10 most active authors and cited authors.

Rank	Most active authors				Most cited authors			
	Author	Publications	TGCS	Average citations per paper	Cocited author	Publications	TGCS	Average citations per paper
1st	Sadoshima, Junichi	10	2498	249.8	Sadoshima, Junichi	10	2498	249.8
2nd	Kanamori, Hiromitsu	9	888	98.7	Sciarretta, Sebastiano	7	2028	289.7
3rd	Pang, Shuchao	9	99	11.0	Del re,Dominic P	3	1450	483.3
4th	Yan, Bo	9	99	11.0	Zhai, peiyong	4	1242	310.5
5th	Sun, Dongdong	8	390	48.8	Wang, kun	6	1202	200.3
6th	Sciarretta, Sebastiano	7	2028	289.7	Forte, Maurizio	4	931	232.8
7th	Hill, Joseph A.	7	902	128.9	Frati, Giacomo	4	931	232.8
8th	Takemura, Genzou	7	861	123.0	Liu,cui yun	3	926	308.7
9th	Minatoguchi, Shinya	7	826	118.0	Zhou, Lu yu	3	926	308.7
10th	Watanabe, Takatomo	7	799	114.1	Hill, Joseph A.	7	902	128.9

### 3.7. Analysis of Publications and Cited References

**Figure 8:**
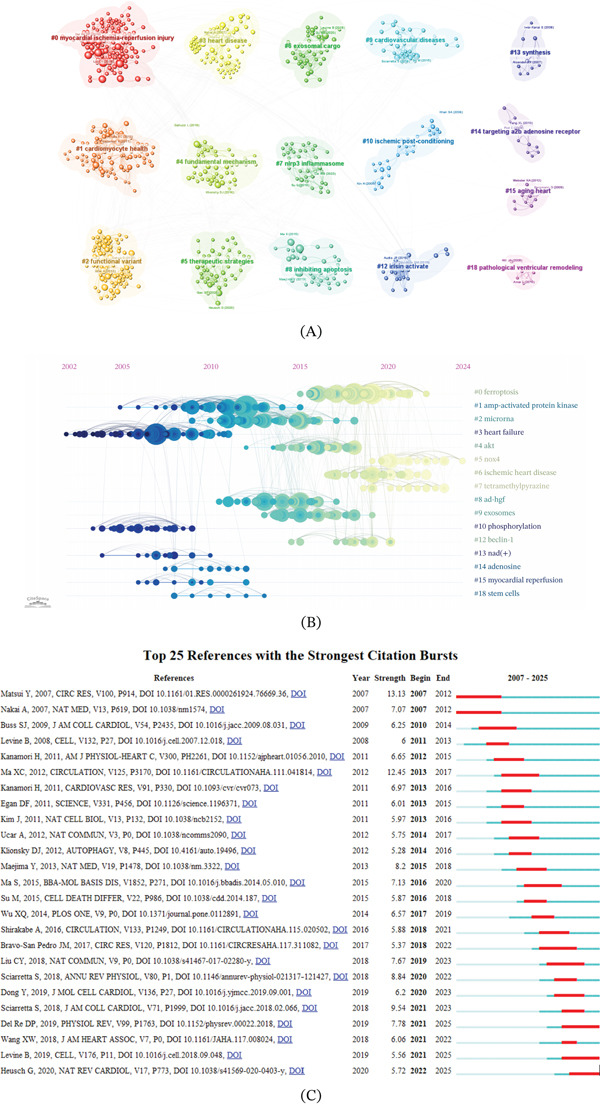
Visualization of references (A) Analysis of cocited reference clustering. (B) The timeline view map of reference cocitation analysis. (C) The Top 25 references with the strongest citation bursts.

To further characterize the most influential literature in this research field, a citation analysis was performed to examine highly cited publications, with review articles and original research analyzed separately (Tables 5 and 6). The results indicate that these two article types exhibit distinct citation patterns and journal distributions. Among review articles, the most highly cited publication was *Current Mechanistic Concepts in Ischemia and Reperfusion Injury* by Wu, Mengyu et al. published in *Cellular Physiology and Biochemistry* (TGCS = 1106; average citations per year = 158.0). Other highly cited reviews were primarily published in journals such as *Nature Reviews Cardiolog* and *Circulation Research*, and generally provided comprehensive overviews of research progress related to myocardial ischemia–reperfusion injury (MIRI) and autophagy‐associated mechanisms. In contrast, the most highly cited original research article was *Mst1 Inhibits Autophagy by Promoting the Interaction Between Beclin1 and Bcl-2* by Maejima, Yasuhiro et al. published in *Nature Medicine* (TGCS = 451; average citations per year = 37.6). Additional highly cited original studies appeared in journals including *Nature Communication* and *Circulation,* and mainly reported experimental observations and molecular regulatory mechanisms of autophagy in the context of MI. Using CiteSpace, we performed keyword co‐occurrence cluster analysis and generated timeline visualizations based on cocitation clustering (Figure 8A,B). Figure 8A displayed 15 distinct color‐coded clusters corresponding to different research themes. By analyzing publication years of references, node size proportionally represents the research activity level of keywords during specific periods, revealing the temporal evolution of research hotspots (Figure 8B). Early‐stage research focused on #3 heart failure, #10 phosphorylation, #13 nad(+), #14 adenosine, #15 myocardial reperfusion, and #18 stem cells. Intermediate‐phase hotspots included #1 amp‐activated protein kinase, #2 microrna, #4 akt, #8 ad‐hgf, #9 exosomes, and #12 beclin‐1. The current frontiers are #0 ferroptosis, #5 n0x4, #6 ischemic heart disease, #7 tetramethylpyrazine, with #0 ferroptosis representing a persistently high‐impact research frontier. Among the 25 most cited references in CiteSpace (Figure 8C), recent hotspots include *Myocardial ischaemia-reperfusion injury and cardioprotection in perspective and Biological Functions of Autophagy Genes A Disease Perspective*. The article with the strongest burst was *Distinct Roles of Autophagy in the Heart During Ischemia and Reperfusion: Roles of AMP-Activated Protein Kinase and Beclin 1 in Mediating Autophagy* (burst strength = 13.13).

**Table 5:** Top 10 cited review articles.

Rank	Title	First author	TGCS	Journal	IF (2025)	Citations per year
1st	Current Mechanistic Concepts in Ischemia and Reperfusion Injury	Wu, Mengyu	1106	Cellular Physiology and Biochemistry	2.0	158.0
2nd	Myocardial Ischaemia‐Reperfusion Injury and Cardioprotection in Perspective	Heusch, Gerd	947	Nature Reviews Cardiology	44.2	189.4
3rd	Fundamental Mechanisms of Regulated Cell Death and Implications for Heart Disease	Del Re, Dominic P.	784	Physiological Reviews	29.9	130.7
4th	Physiological and Pathological Cardiac Hypertrophy	Shimizu, I.	738	Journal of Molecular and Cellular Cardiology	5.2	82.0
5th	Autophagy and Mitophagy in Cardiovascular Disease	Bravo‐San Pedro, J.M.	706	Circulation Research	16.5	88.3
6th	Signaling Pathways and Targeted Therapy for Myocardial Infarction	Zhang, Q.	579	Signal Transduction and Targeted Therapy	40.8	193.0
7th	Ferroptosis as a Novel Therapeutic Target for Cardiovascular Disease	Wu, Xinguang	475	Theranostics	13.3	118.8
8th	Cardiomyocyte Death: Mechanisms and Translational Implications	Chiong, Mario	394	Cell Death & Disease	9.6	28.1
9th	Nrf2 at the Heart of Oxidative Stress and Cardiac Protection	Chen, Q.M.	392	Physiological Genomics	2.5	56.0
10th	Mammalian Target of Rapamycin Signaling in Cardiac Physiology and Disease	Sciarretta, Sebastiano	383	Circulation Research	16.5	34.8

**Table 6:** Top cited original research articles.

Rank	Title	First author	TGCS	Journal	IF (2025)	Citations per year
1st	Mst1 Inhibits Autophagy by Promoting the Interaction Between Beclin1 and Bcl‐2	Maejima, Yasuhiro	451	Nature Medicine	58.7	37.6
2nd	Activated Platelets Present High Mobility Group Box 1 to Neutrophils, Inducing Autophagy and Promoting the Extrusion of Neutrophil Extracellular Traps	Maugeri, N.	450	Journal of Thrombosis and Haemostasis	5.5	40.9
3rd	Improvement of Cardiac Functions by Chronic Metformin Treatment Is Associated With Enhanced Cardiac Autophagy in Diabetic OVE26 Mice	Xie, Zhongxin	441	Diabetes	7.5	31.5
4th	Parkin Protein Deficiency Exacerbates Cardiac Injury and Reduces Survival Following Myocardial Infarction	Kubli, Dieter A.	394	Journal of Biological Chemistry	4.0	32.8
5th	APF IncRNA Regulates Autophagy and Myocardial Infarction by Targeting miR‐188‐3p	Wang, Kun	387	Nature Communications	12.1	38.7
6th	LncRNA CAIF Inhibits Autophagy and Attenuates Myocardial Infarction by Blocking p53‐Mediated Myocardin Transcription	Liu, Cui Yun	328	Nature Communications	15.7	46.9
7th	Mechanisms of Cell Death in Heart Disease	Konstantinidis, K.	319	Arteriosclerosis, Thrombosis, and Vascular Biology	5.5	24.5
8th	Oxidative Stress Stimulates Autophagic Flux During Ischemia/Reperfusion	Hariharan, N.	313	Antioxidants & Redox Signaling	6.1	22.4
9th	Histone Deacetylase Inhibition Blunts Ischemia/Reperfusion Injury by Inducing Cardiomyocyte Autophagy	Xie, Min	289	Circulation	38.6	26.3
10th	FoxO Transcription Factors Promote Cardiomyocyte Survival upon Induction of Oxidative Stress	Sengupta, Anirban	284	Journal of Biological Chemistry	4.0	20.3

### 3.8. Keyword and Thematic Analysis

**Figure 9:**
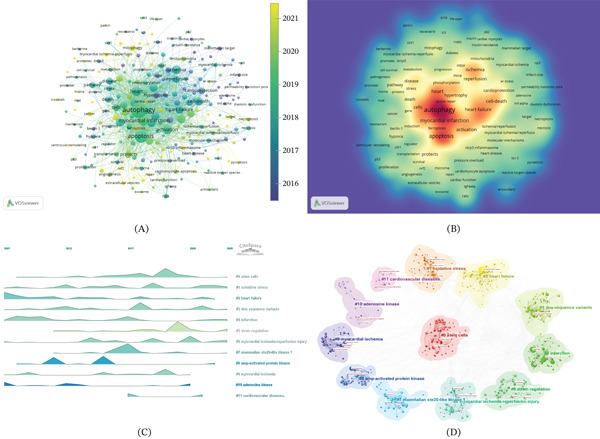
Visualization of keywords. (A) The overlay visualization map of keywords. (B) Visualization of keywords. (C) The keyword clustering volcano maps. (D) The cluster view map of keywords analysis.

This study identified 2505 keywords through comprehensive analysis. Figure 9A (generated by VOSviewer) and Table 7 show the keyword co‐occurrence network analysis, whereas Figure 9B displays the corresponding keyword density visualization. The five most frequently occurring terms were autophagy (*N* = 419), apoptosis (*N* = 236), myocardial infarction (*N* = 138), heart (*N* = 124), and activation (*N* = 104). The volcano plot analysis (Figure 9C) revealed that “oxidative stress,” “infarction,” and “down regulation” currently represent the most prominent research hotspots.

**Table 7:** Top 20 keywords.

Rank	Keyword	Occurrence	Rank	Keyword	Occurrence
1st	Autophagy	419	11th	Inhibition	79
2nd	Apoptosis	236	12th	Ischemia/reperfusion injury	79
3rd	Myocardial infarction	138	13th	Expression	78
4th	Heart	124	14th	Cardiomyocytes	73
5th	Activation	104	15th	Ischemia‐reperfusion injury	67
6th	Oxidative stress	98	16th	Injury	67
7th	Ischemia	96	17th	Inflammation	65
8th	Heart‐failure	91	18th	Mechanisms	62
9th	Cell‐death	85	19th	Protects	62
10th	Myocardial‐infarction	83	20th	Cells	62

**Figure 10:**
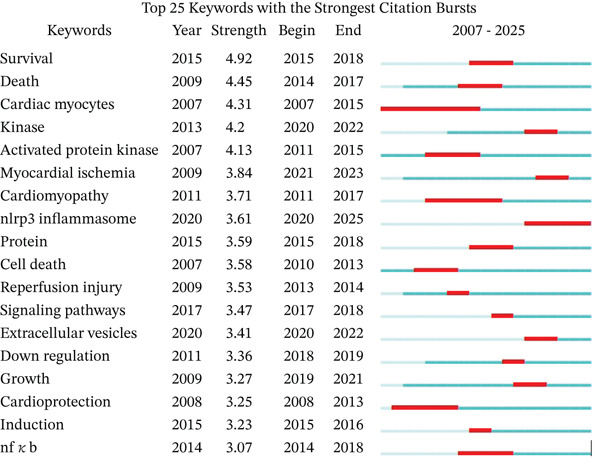
The Top 25 keywords with the strongest citation bursts.

Further analysis using CiteSpace generated a network of keywords occurring at least 25 times, which formed 12 distinct clusters as visualized in Figure 9D. These clusters included #0 stem cells, #1 oxidative stress, #2 heart failure, #3 dna sequence variants, #4 infarction, #5 down regulation, #6 myocardial ischemia‐reperfusion injury, #7 mammalian ste20‐like kinase 1, #8 amp‐activated protein kinase, #9 myocardial ischemia, #10 adenosine kinase, #11 cardiovascular diseases. Subsequent volcano plot analysis identified #0 stem cells as demonstrating the strongest temporal continuity. As shown in Figure 9C and keyword burst detection analysis (Figure 10), both #6 myocardial ischemia‐reperfusion injury and NLRP3 inflammasome emerged as the most recent research hotspots, indicating current directions in the field.

## 4. Discussion

### 4.1. General Information

This study conducted a systematic bibliometric analysis of 649 publications on autophagy in MI published between 2007 and 2025. Owing to its dual regulatory role in cardiomyocyte survival and its considerable translational potential, autophagy has emerged as a prominent research focus in cardiovascular medicine. The temporal analysis identified three characteristic developmental phases: an initial exploratory phase (2007–2016), marked by steady annual growth that gradually established the conceptual and methodological foundations; a rapid expansion phase (2017–2021), characterized by a pronounced increase in research output and peaking at 69 publications in 2021; and a recent phase (2022–2025) showing year‐to‐year fluctuations in publication numbers, reflecting a transition toward more focused and specialized research directions rather than a sustained increase or decline.

### 4.2. Geographic and Institutional Contributions

Research on autophagy in MI involves contributions from 45 countries and regions, indicating substantial global engagement. China showed the highest publication output, followed by the United States, and both countries also ranked highly in TLCS and TGCS, reflecting their strong overall research presence. In contrast, some countries with fewer publications, such as Japan, achieved comparatively high citation impact per paper, indicating that the impact of these national studies is greater. Collaboration analysis shows that China and the United States function as key hubs in the international network, whereas European countries contribute through diverse and cross‐regional partnerships. Over time, publication activity has increased notably in China, alongside sustained contributions from North America and Europe, highlighting an evolving but increasingly interconnected research landscape. All of the five most productive institutions were based in China, reflecting the strong and sustained research activity in this country, with Harbin Medical University contributing the highest number of publications. In contrast, several institutions with relatively lower publication output, such as Rutgers New Jersey Medical School and IRCCS Neuromed, have demonstrated a high level of citation impact. This pattern likely reflects high‐impact research projects and stable collaborations with internationally renowned research institutions, as well as contributions from established investigators in cardiac autophagy research, including groups led by Sciarretta, Sebastiano. Network analysis further indicates that, despite increasing interinstitutional collaboration, research activities remain organized into relatively discrete clusters, with limited cross‐cluster integration. Strengthening collaboration across institutions and regions may therefore facilitate broader knowledge exchange and support the development of more integrated research approaches in autophagy‐related MI.

### 4.3. Journal Analysis and Interdisciplinary Convergence

Among the most active journals in this field, these journals maintain high academic quality while sustaining substantial output, indicating that they serve as primary platforms for autophagy related MI research. *Circulation* demonstrated the highest citation frequency, suggesting its preeminent status in this field. In addition, journal overlay analysis suggests that autophagy‐related MI studies span multiple disciplinary domains, including molecular biology, pharmacology, and chemistry, with emerging intersections involving materials science. Recent studies have reported the application of nanomaterial‐based delivery systems to modulate autophagy‐related pathways in MI models, highlighting the growing contribution of cross‐disciplinary approaches to mechanistic and therapeutic exploration. For example, cerium oxide nanoparticles have been reported to attenuate MIRI by reducing mitochondrial oxidative stress and modulating autophagy‐related pathways, highlighting the potential of redox‐active nanomaterials in mitophagy‐associated cardioprotection [14].

### 4.4. Author Productivity and Influence

Among the Top 5 most productive authors in this field, three were affiliated with Chinese institutions and two with Japanese institutions. Japanese authors demonstrate high publication output and strong collaborative networks, making substantial contributions to the field, particularly Sciarretta, Sebastiano and Kanamori, Hiromitsu, whose pioneering investigations have included seminal contributions to elucidating the dual role of autophagy in MIRI [15] and its relationship with cardiac fibrosis [16]. Author clusters exhibit relative isolation, suggesting limited cross‐group collaboration. Enhanced scholarly exchange between research groups is therefore recommended to foster knowledge integration. In the cocited author network, Mizushima, N. occupies a high centrality position, consistent with the widespread citation of his foundational review Autophagy fights disease through cellular self‐digestion [17], which delineates the molecular machinery and physiological functions of autophagy. This work is frequently cocited across multiple research directions related to cardiovascular and cellular stress biology.

### 4.5. Reference Analysis

Bibliometric analysis indicates that the most influential among the Top 10 highly cited review articles is the 2018 review by Wu, Mengyu [18] published in *Cell Physiology and Biochemistry*. This study systematically dissected the complex mechanisms of MIRI, laying the groundwork for subsequent research. Among the Top 10 highly cited original articles, the paper with the highest impact factor was authored by Maejima et al. [19] in *Nature Medicine*. This research elucidated the MST1‐Beclin1‐Bcl‐2 pathway as a key mechanism linking autophagy inhibition to MIRI, providing theoretical underpinnings for developing novel cardioprotective therapies. Additionally, Zhang et al.′s review published in *Signal Transduction and Targeted Therapies* [20], holds the highest average annual citation count. This review systematically maps key signaling pathway networks involved in MI development and focuses on novel therapeutic strategies targeting these pathways. Moreover, research indicates that review articles typically garner more citations than original research. This is primarily because reviews focus on synthesizing research progress and serving as general references within a field, whereas original research concentrates on specific mechanisms. Although possessing significant scientific value, the latter tends to have a relatively limited citation reach.

### 4.6. Identification of Research Hotspots and Emerging Topics

The current research frontiers and hotspots can be broadly categorized into three aspects through the analysis of reference timelines, keyword clustering, keyword bursts, and qualitative keyword analysis. The following mechanistic subsections are intended as a contextual synthesis of representative and highly cited studies identified through bibliometric analysis. These discussions are not direct outputs of citation or co‐occurrence networks, but are included to provide biological context for the major research themes highlighted by the bibliometric results.

#### 4.6.1. The Dual‐Phase Regulation of Autophagy in MI

The recurrent co‐occurrence of terms related to autophagic flux, cardioprotection, and cell death reflects growing recognition of a dual‐phase role of autophagy during MI progression.

Autophagy plays a dual‐phase role in the pathogenesis of MI [21]. In early ischemia, moderate activation through the AMPK/mTORC1 axis selectively eliminates damaged mitochondria and misfolded proteins, while recycling metabolic substrates to mitigate energy deficiency and preserve cardiomyocyte viability [22]. With prolonged ischemia or reperfusion, autophagy becomes dysregulated [23], characterized by lysosomal dysfunction, ROS accumulation, impaired autophagosome–lysosome fusion, and disrupted flux integrity. When autophagosome formation surpasses degradative capacity, undegraded autophagosomes accumulate within cells, depleting essential components and activating Na^+^/K^+^‐ATPase–dependent autosis, thereby establishing a self‐amplifying cycle of cell death [24]. Notably, due to metabolic heterogeneity and stress tolerance among cardiac cell types, the threshold for autophagy′s shift from protective to detrimental effects varies considerably [5], providing a rationale for cell type–specific regulation. Current research hotspots focus on precisely defining the “key temporal nodes” and molecular switches underlying the transition of autophagy properties in different cell types, as well as on regulating the degree of autophagy, with the ultimate goal of achieving its precise application in MI therapy. For example, in cardiomyocytes, AMPK‐ULK1 activation within 30 min of ischemia promotes protective autophagy [25]. In macrophages, delayed TFEB translocation markedly worsens inflammation. These findings provide important evidence for the precise treatment of MI through the regulation of autophagy with spatiotemporal specificity.

Recent studies indicate that inhibiting CD36 palmitoylation dual‐improves cardiac function by alleviating fatty acid overload and enhancing mitochondrial phagocytosis, suggesting its potential value in MI treatment [26]; macrophage membrane‐coated biomimetic nanoparticles (MM/RESNPs) [27], through precise targeted delivery, significantly improve cardiac function and reduce infarct size in MI mice. These drugs and therapies offer novel avenues for achieving precision treatment of MI, though their application remains subject to further clinical validation.

#### 4.6.2. Mitochondrial Autophagy (Mitophagy) in Cardioprotection

Mitochondrial autophagy (Mitophagy) is a critical therapeutic target in MI [28], due to the reliance of cardiomyocytes on mitochondrial oxidative phosphorylation [29]. Experimental studies show that ischemia impairs the electron transport chain, reduces ATP synthesis, and increases ROS production, leading to energy depletion and oxidative damage [30, 31]. Mitophagy confers cardioprotection through two mechanisms: It selectively removes damaged mitochondria via PINK1‐Parkin pathways, which serve as the primary quality control system [32, 33], and it provides multiple protective effects by maintaining energy metabolism, reducing oxidative stress, and preventing apoptosis through the removal of dysfunctional mitochondria [33, 34]. Intervention studies demonstrate that controlled mitophagy activation improves cardiomyocyte survival, whereas inhibition aggravates energy failure and lipid peroxidation [35]. These findings have driven research on autophagy modulators such as urolithin A [36], which exert cardioprotective effects in ischemia–reperfusion injury by enhancing mitochondrial autophagy and alleviating oxidative stress and ferroptosis. Correspondingly, studies on the regulation of FUNDC1/BNIP3 expression have demonstrated that upregulating FUNDC1 or fine‐tuning BNIP3 can restore impaired mitochondrial autophagy flux, improve myocardial cell survival, and mitigate myocardial injury [37]. Clinical application, however, requires addressing safety concerns, including precise spatiotemporal control of mitophagy activation, tissue‐specific targeting, and minimization of off‐target or long‐term adverse effects [38].

#### 4.6.3. Autophagy‐NLRP Inflammasome Crosstalk in MI

Postinfarction inflammation is initiated when necrotic cells release DAMPs such as mtDNA and ATP, which activate the TLR/NF‐*κ*B pathway to induce inflammasome component expression [39]. Concurrently, mitochondrial damage leads to mtROS leakage, which directly triggers NLRP3 assembly [40]. This cascade drives excessive inflammatory activation, exemplified by NLRP3‐dependent IL‐1*β* release, which serves as a central mechanism of secondary injury [39, 40]. Conventional anti‐inflammatory agents, such as colchicine, show limited efficacy because they cannot target upstream signaling events. Recent studies indicate that autophagy exerts a dual inhibitory effect on NLRP3 activation. Mitophagy selectively removes damaged mitochondria, thereby reducing mtROS and mtDNA leakage [41]. In parallel, autophagosomes mediate p62/SQSTM1‐dependent lysosomal degradation of NLRP3, ASC, and pro‐IL‐1*β* [42, 43]. evidence indicates that autophagy deficiency (e.g., Atg5 knockout) exacerbates NLRP3 activation [44], whereas pharmacological activation of autophagy (e.g., rapamycin) or inhibition of NLRP3 (e.g., MCC950) attenuates myocardial injury [45]. These findings support a shift in therapeutic strategies from single‐target approaches to dual‐target interventions, such as the combination of autophagy inducers with NLRP3 inhibitors, or precision delivery systems including TFEB‐targeted nanoparticles [46] and AAV9‐mediated TFEB gene therapy [47]. Pharmacologically, autophagy activation (e.g., by rapamycin) can enhance autophagic flux by inhibiting the mTOR signaling pathway, promoting the clearance of damaged mitochondria and abnormal proteins, thereby reducing cardiomyocyte death and improving myocardial remodeling. Alternatively, inhibition of NLRP3 (e.g., via MCC950 to suppress NLRP3 and its downstream inflammatory factors) can attenuate inflammatory responses and myocardial fibrosis, consequently mitigating myocardial injury [48]. This transition highlights the movement from nonspecific anti‐inflammatory approaches toward precise modulation of the autophagy–inflammation network.

## 5. Limitation

It should be noted that this study has several limitations. First, as a bibliometric analysis, it primarily relies on automated tools for data processing. Although such tools are efficient for large‐scale literature analysis, their reliance on predefined algorithms may introduce biases in complex topic identification and interdisciplinary classification. Second, although both WoSCC and Scopus were included to enhance coverage, inherent database‐specific biases in WoSCC and Scopus may influence the presentation of certain research findings. Therefore, the merged dataset captures major research trends and collaboration patterns but does not constitute a fully comprehensive global literature corpus. Additionally, due to database update mechanisms, publications appearing after the search cutoff date were not recorded, resulting in results reflecting literature up to that time point. Finally, this study is constrained by the inherent limitations of descriptive bibliometric analysis. The identification of influential authors, articles, and journals primarily relies on citation‐based metrics, which may be affected by structural biases such as self‐citations, publication time effects, and database‐specific coverage.

## 6. Conclusion

This bibliometric analysis systematically evaluated MI‐autophagy research from 2007 to 2025 using multiple analytical tools (HistCite Pro, CiteSpace, VOSviewer, and SCImago Graphica). The analysis demonstrates that the field has reached a mature developmental stage, with China and the United States emerging as the most productive contributors, whereas international collaborations show potential for expansion. Key research hotspots include “autophagy,” “myocardial infarction,” and “inflammation,” with particular emphasis on mitophagy‐NLRP3 inflammasome crosstalk as a promising therapeutic target.

## Author Contributions

All authors contributed to the study conception and design. Xiao‐lin Li: writing—review and editing, writing—original draft, and visualization. Jin‐wen Wu: writing—visualization and validation. Zeng‐dai‐quan Ke: writing—methodology, investigation, and formal analysis. Yuan‐li Hu: writing—review and editing and data curation. Hang Jiang: data curation, validation, and methodology. Ming‐tai Chen: supervision and project administration. Han‐yu Hu: resources and methodology. Zhong‐jing Hu: writing—review and editing and validation. Yuan‐yuan Li: software and resources. Gang Luo: validation and methodology. Meng‐nan Liu: writing—review and editing, methodology, funding acquisition, and conceptualization. Xiao‐lin Li, Jin‐wen Wu, and Zeng‐dai‐quan Ke have contributed to the work equally and should be regarded as co‐first authors.

## Funding

This work was supported by the National Natural Science Foundation of China (82074378), the Project of Science & Technology Department of Sichuan Province (2026NSFSC1823), Youth Innovation Project of Sichuan Medical Association (Q20250014), 2024 Traditional Chinese Medicine Guangdong Provincial Laboratory Project (HQCML‐C‐2024005), Shenzhen Science and Technology Program (JCYJ20230807094603007, JCYJ20240813152440051), Shenzhen Medical Research Fund (A2403028), and the Project of Southwest Medical University (2024ZXYZX30, 2024ZKZ007, 2024ZXYZX39, 2024ZKY073).

## Disclosure

The funder had no role in the study design, data analysis, or decision to publish. All authors have read and approved the final manuscript and agree to be accountable for the work.

## Ethics Statement

All analyses were based on previous studies and therefore did not require ethical approval or patient consent.

## Conflicts of Interest

The authors declare no conflicts of interest.

## Data Availability

The data are available on request from the authors.
